# Gender Stratified Analyses of the Association of Skinfold Thickness with Hypertension: A Cross-Sectional Study in General Northeastern Chinese Residents

**DOI:** 10.3390/ijerph15122748

**Published:** 2018-12-05

**Authors:** Yuyan Liu, Yongfang Li, Jing He, Ping Ma, Luyang Yu, Quanmei Zheng, Guifan Sun

**Affiliations:** 1 Research Center of Environmental and Non-Communicable Disease, School of Public Health, China Medical University, Shenyang 110122, China; liuyycmu@163.com (Y.L.); liyongfang_17@163.com (Y.L.); zhengqm2006@163.com (Q.Z.); 2 Department of Non-Communicable Disease, Shenhe Center for Disease Control and Prevention, Shenyang 110122, China; jinghe18@126.com (J.H.); 18604100018@163.com (P.M.); ylyshcdc@163.com (L.Y.)

**Keywords:** skinfold thickness, subcutaneous fat, obesity, blood pressure, hypertension

## Abstract

The association of hypertension with skinfold thickness (ST) in adults is not clear. Our study was aimed at finding out the association of hypertension with ST in different gender and obesity categories. This is a cross-sectional study based on 2336 Chinese residents (767 men). Both subscapular skinfold thickness (SST) and tricep skinfold thickness (TST) were examined. We estimated the association of hypertension with per SD increase of SST and TST using multivariable logistic regression analyses in men and women. Six subgroups were stratified using cutoff points of body mass index (BMI) and ST: larger and smaller ST in normal weight (BMI < 24 kg/m^2^), overweight (24 kg/m^2^ ≤ BMI < 28 kg/m^2^) and obesity (BMI ≥ 28 kg/m^2^), respectively. The association of hypertension with ST was only shown in women after adjustment for other risk factors. Among women of the normal weight subgroup, higher prevalence of hypertension was shown in those with larger ST. No difference of the prevalence of hypertension was found between women with larger ST in the normal weight subgroup and those with smaller ST in overweight or obesity subgroups. Our study suggested that even for people with normal weight, it was necessary to monitor the subcutaneous fat using ST for preventing hypertension at least in general Chinese women.

## 1. Introduction

Subcutaneous adipose tissue (SAT) is an important component of human body fat, with a larger percentage of the overall fat in contrast to visceral adipose tissue (VAT) [1,2]. While in recent years the pathophysiology of VAT is always highlighted, the adverse effect of SAT accumulation seems to be ignored [3,4]. Excess SAT can cause insulin resistance and increase the risk of cardiovascular diseases (CVD), among which hypertension is a main metabolic abnormality [1,5,6]. Epidemiological studies indicate that about 75% of hypertension diagnoses are attributed to obesity, and subcutaneous fat should be given sufficient attention [7]. Skinfold thickness (ST) is an easily obtained obesity index and seems like a good way to estimate the subcutaneous fat [8,9,10,11]. Both subscapular skinfold thickness (SST) and tricep skinfold thickness (TST) have been shown to be positively associated with elevated blood pressure (BP) and hypertension in various populations, especially adolescents, while relevant studies from adults are limited [12,13,14]. Additionally, considering that the percentage of SAT is smaller in male bodies than that in females [15], the variation of hypertension risk attributed to SAT may be different in man and women. Gender stratified epidemiological studies are optimal ways to find out the different associations of hypertension with ST in men and women. 

On the other hand, given that body mass index (BMI) is a widely used traditional obesity index, people always detect the obesity and other CVD risks using it [16,17,18]. However, the excess SAT cannot be estimated using BMI, and subcutaneous fat measured using ST is easy to be ignored especially among people with normal weight. Normal weight obesity (NWO), representing a condition characterized by weight within normal limits according to BMI but with a high percentage of body fat, has been shown to be positively associated with the development of several CVD risk factors, including hypertension [19,20]. If the association of ST with hypertension existed as previous findings showed, we hypothesized that people with normal BMI but larger ST (i.e., NWO) could still have a higher prevalence of hypertension compared with those with normal BMI and smaller ST, such potential risk seems more harmful because of easily being ignored.

Therefore, our study is aiming to find out the associations of both elevated BP and hypertension with ST (SST and TST) in men and women, based on a typical population living in the northeast of China; and also find out if higher prevalence of hypertension existed among people with normal BMI and larger ST.

## 2. Materials and Methods

### 2.1. Study Design and Participants

**Figure 1:**
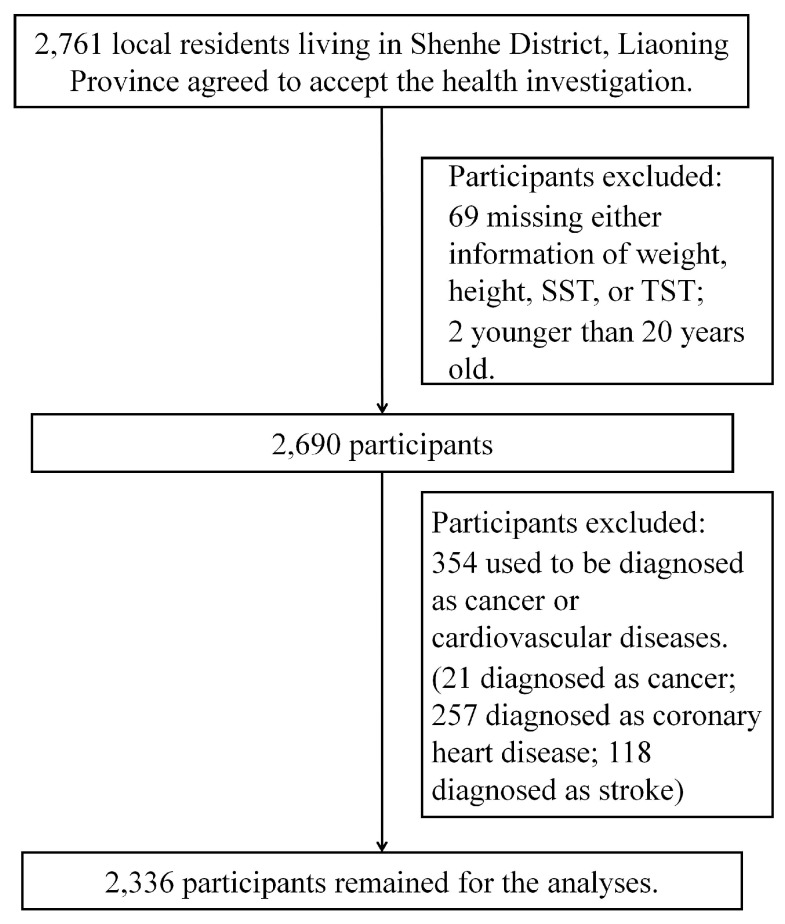
Flowchart for the Present Study.

This is a cross-sectional study based on community-dwelling residents in Shenhe district, Shenyang City, Liaoning Province, China. Details of the methods of enrollment have been reported previously [21,22]. In brief, we randomly selected 2761 local residents living in Shenhe district in 2015, and invited them to complete the questionnaires and undergo the relevant physical examinations and biochemical tests. Of the 2761 participants, 69 participants missing either information of SST or TST were excluded. Two participants younger than 20 years old were then ruled out. Another 354 participants previously diagnosed with cancer or CVD were also excluded (Figure 1). Finally, 2336 participants (767 men and 1569 women) remained for analyses. This study was in accordance with the World Medical Association Declaration of Helsinki-Ethical Principles for Medical Research Involving Human Subjects and was approved by the Ethics Committee of China Medical University (No: CMU62073024). A written informed consent form was obtained from every participant after they had been informed of the objectives, benefits of this study.

### 2.2. Measurements of ST and BMI

We used a skinfold caliper with a constant pressure of 10 g/mm^2^ to measure ST. Firstly, we grasped the fold of skin and underlying SAT 2.0 cm above the place the measurement was to be taken using the thumb and index finger. The jaws of calipers then were placed at the marked level, perpendicular to the skinfold. The measurement was read within 3 s and to the nearest 0.1 mm. SST was measured at approximately at an angle of 45 degree to the lateral side of the body, 20 mm below the tip of the scapula. TST was measured at the halfway between the acromion process and the olecranon process, while the arm is hanging relaxed at the participant’s side. Both SST and TST were measured three times on both sides of the body separately, and averages of 6 measurements were calculated for SST and TST, respectively.

All participants were asked to undergo body weight and height measurements while wearing light clothes without shoes. Body weight and height were measured twice using a vertical weight scale and the metric scale with a standardized protocol, respectively. Both body weight and height were measured to the nearest 0.1 kg and 0.1 cm, respectively. BMI was then calculated as weight (kg) divided by the square of the height (m). According to the currently used cutoff point of BMI in defining the overweight and obesity in Chinese populations, we defined the overweight as 24 kg/m^2^ ≤ BMI < 28 kg/m^2^ and obesity as BMI ≥ 28 kg/m^2^ [23]. 

### 2.3. BP Measurement and the Definition of Hypertension

BP was measured using a standard mercury-column sphygmomanometer with an appropriate adult upper arm cuff size after 15 min of rest in the sitting position based on the standardized procedural guideline [24]. Systolic blood pressure (SBP) and diastolic blood pressure (DBP) were determined by the first and the fifth Korotkoff sounds. We measured BP on both right and left arm firstly, and the arm with higher blood pressure would be selected to obtain three consecutive measurements with a time interval of at least two minutes [25]. Then, the average on the selected arm would be recorded as the final BP. Before measuring, participants were also need to be asked for not doing following behaviors: drinking alcohol, tea or coffee; smoking; or taking any exercise for at least 30 min before measuring BP. In this study, hypertension was defined as SBP/DBP ≥ 140/90 mmHg or anti-hypertensive medication use. 

### 2.4. Other Risk Factor Measurements

All participants were asked for permission to collect a blood sample after an overnight fast of longer than 8 h. The concentrations of total cholesterol (TC), triglycerides (TG), high density lipid cholesterol (HDL-C), and fasting glucose were examined using a Mindray BS 380 Autoanalyzer (Mindray Ltd., Shenzhen, China) at the local community health service center. All assays were performed according to the manufacturer’s instructions. In addition, self-administered questionnaires were also used to obtain information on demographic characteristics, medical history, medication use, smoking habits, and other pertinent factors. Trained staff members confirmed the reported information with each participant. In this study, smoking status was categorized as current smokers (smoking during the last one year or quit smoking for less than six months), past smokers (quit smoking for more than six months), and never. Drinking status were categorized as current drinkers (at least twice per week for men and once per week for women), past drinkers (quit drinking for more than six months), and never.

### 2.5. Statistical Analysis

All continuous variables were presented as mean and standard deviation (SD). Categorical variables were presented as percentages. Student t test and chi-square test were respectively used to estimate the difference of continuous and categorical variables between men and women. Both men and women were also stratified into three age subgroups (<40 years old, 40–65 years old and ≥65 years old) respectively, and *p* values for trends were given. 

Multivariable linear regression analyses were used to estimate the crude and adjusted association of BP (SBP and DBP) with per SD increase of ST (SST and TST) in men and women without any anti-hypertensive medication use respectively. Besides the unadjusted model and age-adjusted model, multi-adjusted model (multivariable adjusted model) was also established, which was adjusted for both life-style (smoking and alcohol drinking) and metabolic risk factors (fasting glucose, TG, TC and HDL-C) in addition to age-adjusted model. Odds ratio (OR) and 95% confidence interval (95% CI) were used to estimate the ratio of hypertension per SD increase of ST (SST and TST) using multivariable logistic regression analyses in men and women. Same regression models (unadjusted model, age-adjusted model, and multi-adjusted model) were used. SST and TST were separately analyzed in all statistical analyses. The interactions of gender on associations of ST with both hypertension and BP were tested by adding a product term (gender × ST) in regression models. Same analyses were respectively repeated in three age subgroups in both men and women, and interactions of age subgroups were tested by adding a product term (age groups × ST).

Since there is no generally accepted cutoff point for ST, we thereafter divided men and women into two subgroups using medians of SST and TST as the cutoff points. BP and the prevalence of hypertension of different ST subgroups were estimated, and multi-adjusted ORs of hypertension relative to those with small ST were given. 

Both men and women were further divided into six subgroups using both cutoff points of BMI and ST (smaller and larger ST in the normal weight, overweight, and obesity subgroups, respectively). We hypothesized that BP and the prevalence of hypertension in participants with only larger ST was higher than those with normal weight and smaller ST, but not lower than those with only overweight or obesity. Therefore, BP and the prevalence of hypertension of participants with larger ST in the normal weight subgroup were compared to that of other five subgroups. Multi-adjusted ORs of hypertension relative to participants with larger ST in the normal weight subgroup were given. All statistical analyses were completed using SAS 9.4 (SAS Institute, Inc., Cary, NC, USA). *p*-Value < 0.05 was considered as statistical significance.

## 3. Results

### 3.1. General Characteristics

Characteristics of the 2336 participants are shown in Table 1 as mean ± SD and percentages. No difference of SST or TST was found between men and women, and men had higher BP and prevalence of hypertension than women. Age stratified results showed that participants in older age subgroups tended to have larger ST, higher BP and higher prevalence of hypertension. However, trends of SST and TST among the three age subgroups were statistically significant only in women (Table S1).

### 3.2. Associations of ST with BP and Hypertension in Men and Women

Table 2 shows that SBP was independently associated with both SST and TST in women, and the increase of SBP per SD of SST and TST were 2.01 mmHg and 1.39 mmHg. In men, SBP was only associated with SST in multi-adjusted models. For DBP, the association with SST in women was only statistically significant (0.91 mmHg increase per SD of SST). Crude and adjusted associations of hypertension with per SD increase of SST and TST were shown in Table 3. In unadjusted and age-adjusted models, both SST and TST were positively associated with hypertension in women, and only SST was associated with hypertension after adjustment for age in men. In multi-adjusted model, the associations of hypertension with both SST and TST remained statistically significant in women (OR = 1.58; 95% CI: 1.40, 1.79 for SST, and OR = 1.50; 95% CI: 1.33, 1.70 for TST). No association of hypertension with either SST or TST was found in men. Full model results of BP and hypertension were shown in Tables S2–S4, and age showed an independent association with both BP and hypertension in men and women. No interaction on the association of BP with ST was significant in multi-adjusted models. However, the interaction of gender on associations of both SST and TST with hypertension was found. Compared to TST, associations of BP and hypertension with SST were stronger in both men and women. 

Age stratified analyses showed that after adjustment for all risk factors, only associations of SBP with SST and TST were statistically significant in men of 40–65 years old (Table S5), and women of >40 years old (Table S6). No association of hypertension with SST and TST was found in either age subgroup of men in multi-adjusted model (Table S7), and associations were only found in women of >40 years old (Table S8). The interaction of age subgroups on association of BP and hypertension with SST and TST was not statistically significant in both men and women.

### 3.3. ST Stratified Analyses in Men and Women

In men, the medians of SST and TST were 24.0 mm and 25.0 mm, respectively. In women, the medians of SST and TST were 23.0 mm and 24.0 mm, respectively. ST stratified analyses showed that in women both BP and the prevalence of hypertension in the larger ST subgroup were higher than those in the smaller ST subgroup, and the differences were statistically significant. Statistically significant difference was only shown in SBP of SST subgroups in men (Table S9).

### 3.4. Prevalence of Hypertension in Subgroups Stratified Using BMI and ST

Analyses stratified using both BMI and SST showed that in men of the normal weight subgroup, there was no difference of BP or prevalence of hypertension between men with larger SST and smaller SST (Table 4). Compared to men with larger SST in the normal weight subgroup, both BP and the prevalence of hypertension were larger in the overweight and obesity subgroup regardless of SST. For women of the normal overweight subgroup, participants with larger SST had higher BP and prevalence of hypertension than those with smaller SST, and the multi-adjusted OR of hypertension relative to the former subgroup was statistically significant (OR = 0.65; 95% CI: 0.46, 0.90). No statistically significant difference of BP or the prevalence of hypertension was found between women with larger SST in the normal overweight subgroup and those with smaller SST in the overweight and obesity subgroups. Consistent results were showed in analyses stratified using BMI and TST (Table 5). Although in normal weight subgroup women with larger TST had higher prevalence of hypertension than those with smaller TST, the multi-adjusted OR of hypertension relative to the former subgroup was not significant.

## 4. Discussion

In summary, we found that SBP was positively associated with both SST and TST in men and women (Table 2), and the associations of hypertension with SST and TST were only found in women after adjustment for other risk factors (Table 3). Compared to men, the associations of hypertension with both SST and TST were stronger in women. Since SAT accounts for larger percentage of the overall adipose tissue accumulation in human bodies, we thought that the pathophysiology of SAT should not be underestimated [5,26,27]. Currently, several image methods such as abdominal computed tomography, and overall body dual-energy X-ray absorptiometry emerged [28,29]. Although the distribution of adipose tissue and percentage of body fat can be directly and accurately estimated using these methods, disadvantages including expensive costs, complicated operations, and harmful effects on human bodies restrict them to be generally used in populations. In contrast, both SST and TST make it easier for different populations to estimate the subcutaneous fat by themselves. Studies from adolescent populations have shown the association of hypertension with ST, while findings from adults were still limited [13,14,30]. There is a 22-year cohort study from Chinese population showing that the TST larger than 50th percentile was a risk factor of hypertension in both men and women, which was partially consistent with our results [31]. Our findings, as well as previous studies revealed that ST (SST and TST) could be generally used as a good obesity index in detecting hypertension. By using these easily obtained obesity indices, people can monitor their subcutaneous fat more frequently, and the awareness of preventing hypertension and other CVD risks could be enhanced. 

Our results showed that in both men and women, SST was stronger associated with BP and hypertension than TST. In published results, only one ST measurement or combining both values of SST and TST were analyzed [14,30,31]. We thought our study is the first study comparing the strengths of association of both SST and TST with hypertension using standardized regression coefficients. This finding implied that in our specific populations, SST could be a better choice in the clinical utilities. It is possible that results from other populations would be inconsistent with ours, and variations of populations could perhaps partially explain the inconsistency. Additionally, given that the percentage of SAT is larger in female bodies than that in male [32,33], the variation of hypertension risk attributed to SAT perhaps becomes attenuated in men. This could partially explain the stronger associations of ST with both hypertension and elevated BP in women. Our results implied that ST was probably more useful in women for measuring the subcutaneous fat and detecting hypertension. 

According to the medians of SST and TST in men and women, we divided participants into two subgroups. However, the statistically significant differences of BP and the prevalence of hypertension between ST stratified subgroups were only found in women (Table S9). Currently in China, the measure of ST is still far from being generally used compared to other obesity indices, such as BMI and waist circumference, and there is not a normal cutoff point of SST or TST for general Chinese adults published so far. What we found could add to the literature finding out the optimal cutoff points of ST in Chinese populations. 

In women of the normal weight subgroup, both BP and the prevalence of hypertension of participants with larger ST were higher than those with smaller ST, and the multi-adjusted OR of hypertension relative to the former subgroup was statistically significant (Table 4 and Table 5). People always pay more attention to BMI, and ignore the accumulation of SAT, especially for those in normal weight. However, according to our results, people with both larger ST and normal BMI (i.e., NWO) could still have a higher prevalence of hypertension at least in women. What we found was consistent with previous studies, in which the associations of NWO with various metabolic abnormalities relevant to CVD were shown [34,35,36,37]. However, if people in NWO had similar prevalence of abnormalities with those in overweight or obesity was not clear in these studies [28]. Our results showed that the difference of BP or prevalence of hypertension between women with larger ST in the normal overweight subgroup and those with smaller ST in overweight and obesity subgroups was not statistically significant, and neither was the multi-adjusted OR, which implied that in terms of the prevalence of hypertension, there was no difference between participants with only larger ST and those with only overweight or obesity. This result, we thought, could provide some new views in preventing obesity and hypertension. NWO could not be overlooked for people with normal weight, and the routine measurements of SST and TST seemed good ways to monitor the obesity situation and prevent the hypertension.

Several limitations should be considered when interpreting our results. Compared to women, the small sample size of men perhaps resulted in the attenuated association of ST with both elevated BP and hypertension. On the other hand, participants in our study were concentrated on the middle (40–65 years old) and old age (≥65 years old), which resulted in the statistically significant associations of ST with both BP and hypertension were only found in these two subgroups. However, no interaction of age subgroups was found. Secondly, because of the observational and cross-sectional nature of this study, we were unable to prove the causality between ST (SST and TST) and hypertension, and longitudinal following-up study should be continued. Likewise, only local residents were recruited from a single area in this study. Therefore, conclusions from this study may be different in other populations with various life-styles. Another limitation was that several life-style factors correlated with hypertension, such as dietary intakes and physical activity were not included in our study. Hypertension is a multifactorial and multigenic disease, both genetic factors and life-style factors should be considered in further relevant studies. One of strengths in our study was that we randomly selected a community-based sample with a broad age range, which increased the generalizability to general Chinese residents. Secondly, we compared strengths of associations of BP and hypertension with SST and TST using a standardized coefficient, and the results could provide some reference in the utility of ST. Use of standardized protocols in assessing exposures (SST and TST) and outcomes (BP and hypertension) is another strength of our study. 

## 5. Conclusions

Both SST and TST were positively associated with hypertension in women. In women of the normal weight subgroup, the prevalence of hypertension in those with larger ST was higher than those with small ST. There was no difference of the prevalence of hypertension between women with only larger ST and those with only overweight or obesity. Our study suggested that even for people with normal BMI, it was also necessary to monitor the subcutaneous fat using ST for preventing hypertension at least in general Chinese women.

## Figures and Tables

**Table 1:** Baseline characteristics of participants from Shenhe District, Liaoning Province, China (2336 participants, aged 20 to 94 years old, examined in 2015).

	Men (*n* = 767)	Women (*n* = 1569)
Age (years old)	58.9 ± 11.2 *	57.7 ± 11.5
Weight (kg)	73.3 ± 1.09 **	62.0 ± 9.0
Height (cm)	172.1 ± 5.1 **	160.2 ± 4.7
BMI (kg/m^2^)	24.7 ± 3.4 **	24.2 ± 3.3
SST (mm)	24.1 ± 9.3	23.7 ± 8.7
TST (mm)	25.7 ± 11.2	24.8 ± 9.9
SBP (mmHg)	131.8 ± 12.1 **	127.5 ± 14.5
DBP (mmHg)	81.5 ± 8.4 **	78.0 ± 8.5
Fasting glucose (mmol/L)	5.9 ± 1.8	5.7 ± 2.8
TG (mmol/L)	2.2 ± 6.4	1.8 ± 1.1
TC (mmol/L)	5.0 ± 1.0 **	5.3 ± 1.2
HDL-C (mmol/L)	1.4 ± 0.4 **	1.5 ± 0.5
Hypertension (%)	42.6 *	35.6
Antihypertensive medication (%)	27.8 *	23.1
Smoking (%)		
Current	24.4 **	1.5
Past	16.1	2.0
Never	59.5	96.5
Drinking (%)		
Current	51.4 **	14.2
Past	2.7	0.8
Never	45.9	85.0

Values are presented as mean ± SD, or %. Abbreviations: BMI: body mass index; SST: subscapular skinfold thickness; TST: tricep skinfold thickness; SBP: systolic blood pressure; DBP: diastolic blood pressure; TG: triglycerides; TC: total cholesterol; HDL-C: high density lipid cholesterol. *: *p*-value < 0.05; **: *p*-value < 0.001.

**Table 2:** Crude and adjusted associations of blood pressure with per SD increase of skinfold thickness (1760 participants without anti-hypertensive medication, aged 20 to 94 years old, examined in 2015).

	Men (*n* = 554)			Women (*n* = 1206)			*p* for Interaction
	BP (mmHg)	95% CI	*p*-Value	BP (mmHg)	95% CI	*p*-Value	
SBP							
Unadjusted Model							
SST	1.31	0.49, 2.12	0.002	2.71	1.93, 3.50	<0.001	0.025
TST	1.02	0.23, 1.80	0.011	2.05	1.25, 2.86	<0.001	0.096
Age-Adjusted Model							
SST	1.25	0.44, 2.06	0.002	2.12	1.39, 2.85	<0.001	0.072
TST	0.90	0.12, 1.68	0.023	1.48	0.73, 2.23	<0.001	0.139
Multi-Adjusted Model							
SST	1.16	0.34, 1.99	0.006	2.01	1.26, 2.75	<0.001	0.088
TST	0.83	0.04, 1.62	0.040	1.39	0.62, 2.16	<0.001	0.200
DBP							
Unadjusted Model							
SST	0.35	−0.26, 0.95	0.258	1.31	0.85, 1.77	<0.001	0.015
TST	0.17	−0.41, 0.75	0.570	0.82	0.35, 1.30	<0.001	0.092
Age-Adjusted Model							
SST	0.35	−0.25, 0.95	0.256	1.08	0.63, 1.53	<0.001	0.029
TST	0.17	−0.41, 0.73	0.563	0.60	0.13, 1.06	0.012	0.117
Multi-Adjusted Model							
SST	0.27	−0.35, 0.89	0.390	0.91	0.46, 1.37	<0.001	0.057
TST	0.08	−0.51, 0.67	0.785	0.44	−0.03, 0.91	0.064	0.213

SST and TST were analyzed in separate regression models. Multi-adjusted model: adjusted for smoking, alcohol drinking, fasting glucose, TG, TC, and HDL-C in addition to age-adjusted model. Abbreviations: BP: blood pressure; 95% CI: 95% confidence interval; SBP: systolic blood pressure; DBP: diastolic blood pressure; SST: subscapular skinfold thickness; TST: tricep skinfold thickness.

**Table 3:** Crude and adjusted associations of hypertension with per SD increase of skinfold thickness (2336 participants, aged 20 to 94 years old, examined in 2015).

	Men (*n* = 767)			Women (*n* = 1569)			*p* for Interaction
	OR	95% CI	*p*-Value	OR	95% CI	*p*-Value	
Unadjusted Model							
SST	1.18	1.03, 1.36	0.018	1.62	1.45, 1.82	<0.001	<0.001
TST	1.10	0.96, 1.25	0.175	1.53	1.37, 1.72	<0.001	<0.001
Age-Adjusted Model							
SST	1.18	1.03, 1.36	0.022	1.59	1.41, 1.80	<0.001	0.003
TST	1.08	0.94, 1.24	0.278	1.51	1.34, 1.71	<0.001	<0.001
Multi-Adjusted Model							
SST	1.15	0.99, 1.33	0.064	1.58	1.40, 1.79	<0.001	0.003
TST	1.05	0.92, 1.21	0.492	1.50	1.33, 1.70	<0.001	<0.001

SST and TST were analyzed in separate regression models. Multi-adjusted model: adjusted for smoking, alcohol drinking, fasting glucose, TG, TC, and HDL-C in addition to age-adjusted model. Abbreviations: OR: odds ratio; 95% CI: 95% confidence interval; SST: subscapular skinfold thickness; TST: tricep skinfold thickness.

**Table 4:** Blood pressure and hypertension in different body mass index and subscapular skinfold thickness stratified subgroups (2336 participants, aged 20 to 94 years old, examined in 2015).

	Total Number	SBP (mmHg)	DBP (mmHg)	Hypertension (%)	OR (95% CI)
Men	767				
Normal weight	330				
SST < 24.0 mm	179	126.0 ± 10.2 *	78.0 ± 6.8	31.8	0.94 (0.58, 1.52)
SST ≥ 24.0 mm	151	129.6 ± 9.6	79.3 ± 7.3	35.1	Ref.
Overweight	345				
SST < 24.0 mm	169	129.4 ± 8.6	80.5 ± 7.2	43.2	1.62 (1.01, 2.61) *
SST ≥ 24.0 mm	176	130.0 ± 10.4	80.5 ± 7.7	50.0*	2.04 (1.28, 3.27) *
Obesity	92				
SST < 24.0 mm	29	130.4 ± 13.1	84.6 ± 13.4 *	72.4 **	7.01 (2.81, 17.51) **
SST ≥ 24.0 mm	63	133.2 ± 12.7	82.2 ± 6.1	55.6 *	2.92 (1.55, 5.52) *
Women	1569				
Normal weight	787				
SST < 23.0 mm	444	119.8 ± 13.0 *	74.0 ± 7.7 *	21.9 **	0.65 (0.46, 0.90) *
SST ≥ 23.0 mm	343	124.9 ± 15.2	76.9 ± 8.1	33.8	Ref.
Overweight	610				
SST < 23.0 mm	255	125.6 ± 11.0	77.4 ± 6.2	32.6	0.96 (0.67, 1.39)
SST ≥ 23.0 mm	355	128.2 ± 12.9 *	78.6 ± 8.4	47.0 **	1.74 (1.26, 2.41) **
Obesity	172				
SST < 23.0 mm	48	126.1 ± 13.3	77.7 ± 10.7	43.8	1.61 (0.84, 3.09)
SST ≥ 23.0 mm	124	130.1 ± 12.1 *	79.4 ± 7.4	59.7 **	2.96 (1.89, 4.65) **

The mean and standard deviation of blood pressure were estimated using participants without anti-hypertensive medication; overweight was defined as body mass index ≥24 kg/m^2^ and <28 kg/m^2^; obesity was defined as body mass index ≥28 kg/m^2^; normal weight subgroups with SST ≥ 24 mm in men and SST ≥ 23 mm in women were the reference groups with which other 5 subgroups were compared in men and women, respectively; SBP and DBP were presented as mean ± SD; odds ratio of hypertension was obtained using multivariable logistic linear regression adjusted for age, smoking, alcohol drinking, fasting glucose, TG, TC, and HDL-C. Abbreviations: SBP: systolic blood pressure; DBP: diastolic blood pressure; OR: odds ratio; 95% CI: 95% confidence interval; SST: subscapular skinfold thickness. *: *p*-value < 0.05; **: *p*-value < 0.001.

**Table 5:** Blood pressure and hypertension in different body mass index and tricep skinfold thickness stratified subgroups (2336 participants, aged 20 to 94 years old, examined in 2015).

	Total Number	SBP (mmHg)	DBP (mmHg)	Hypertension (%)	OR (95% CI)
Men	767				
Normal weight	330				
TST < 25.0 mm	178	127.0 ± 10.0	78.5 ± 6.7	17.1	0.91 (0.56, 1.47)
TST ≥ 25.0 mm	152	128.5 ± 10.1	78.6 ± 7.5	35.5	Ref.
Overweight	345				
TST < 25.0 mm	168	129.4 ± 8.6	80.6 ± 7.4	45.2	1.76 (1.10, 2.82) *
TST ≥ 25.0 mm	177	129.9 ± 10.4	80.4 ± 7.5	48.0 *	1.84 (1.15, 2.93) *
Obesity	92				
TST < 25.0 mm	33	134.4 ± 17.1	85.5 ± 11.3 *	75.8 **	8.01 (3.24, 19.84) **
TST ≥ 25.0 mm	59	131.6 ± 10.4	81.6 ± 7.2	52.5 *	2.60 (1.37, 4.94) *
Women	1569				
Normal weight	787				
TST < 24.0 mm	451	120.9 ± 12.7	74.7 ± 7.8	23.5 *	0.78 (0.56, 1.10)
TST ≥ 24.0 mm	336	123.3 ± 16.0	75.9 ± 8.2	31.9	Ref.
Overweight	610				
TST < 24.0 mm	262	126.7 ± 10.7 *	77.8 ± 6.1 *	34.0	1.09 (0.76, 1.56)
TST ≥ 24.0 mm	348	127.3 ± 13.3 *	78.3 ± 8.5 *	46.3 **	1.91 (1.37, 2.65) **
Obesity	172				
TST < 24.0 mm	53	128.6 ± 14.9	79.9 ± 10.7 *	49.1 *	2.11 (1.12, 3.94) *
TST ≥ 24.0 mm	119	129.0 ± 11.3 *	78.4 ± 7.3	58.0 **	3.09 (1.96, 4.88) **

The mean and standard deviation of blood pressure were estimated using participants without anti-hypertensive medication; overweight was defined as body mass index ≥24 kg/m^2^ and <28 kg/m^2^; obesity was defined as body mass index ≥28 kg/m^2^; normal weight subgroups with TST ≥ 25 mm in men and TST ≥ 24 mm in women were the reference groups with which other 5 subgroups were compared in men and women, respectively; SBP and DBP were presented as mean ± SD; odds ratio of hypertension was obtained using multivariable logistic linear regression adjusted for age, smoking, alcohol drinking, fasting glucose, TG, TC, and HDL-C. Abbreviations: SBP: systolic blood pressure; DBP: diastolic blood pressure; OR: odds ratio; 95% CI: 95% confidence interval; TST: tricep skinfold thickness. *: *p*-value < 0.05; **: *p*-value < 0.001.

## Supplementary Materials

The following are available online at http://www.mdpi.com/1660-4601/15/12/2748/s1, Table S1: baseline characteristics of participants in different age subgroups (2336 participants, aged 20 to 94 years old, examined in 2015), Table S2: associations of systolic blood pressure with all risk factors (1760 participants without anti-hypertensive medication, aged 20 to 94 years old, examined in 2015), Table S3: associations of diastolic blood pressure with all risk factors (1760 participants without anti-hypertensive medication, aged 20 to 94 years old, examined in 2015), Table S4: associations of hypertension with all risk factors (2336 participants without anti-hypertensive medication, aged 20 to 94 years old, examined in 2015), Table S5: crude and adjusted associations of blood pressure with per SD increase of skinfold thickness in men of different age subgroups (554 participants without anti-hypertensive medication, aged 20 to 94 years old, examined in 2015), Table S6: crude and adjusted associations of blood pressure with per SD increase of skinfold thickness in women of different age subgroups (1206 participants without anti-hypertensive medication, aged 20 to 94 years old, examined in 2015), Table S7: crude and adjusted associations of hypertension with per SD increase of skinfold thickness in men of different age subgroups (767 participants, aged 20 to 94 years old, examined in 2015), Table S8: crude and adjusted associations of hypertension with per SD increase of skinfold thickness in men of different age subgroups (1569 participants, aged 20 to 94 years old, examined in 2015), Table S9: blood pressure and hypertension in different skinfold thickness stratified groups (2336 Participants, aged 20 to 94 years old, examined in 2015).
