# Supplementary material for: Gender Stratified Analyses of the Association of Skinfold Thickness with Hypertension: A Cross-Sectional Study in General Northeastern Chinese Residents
Source: Int J Environ Res Public Health. 2018 Dec 5;15(12):2748. doi: 10.3390/ijerph15122748 (PMC6313501; doi:10.3390/ijerph15122748)
Supplement: Supplementary file 1 [file ijerph-15-02748-s001.pdf]

Supplemental Tables

Gender Stratified Analyses of the Association of Skinfold Thickness with Hypertension: A Cross-Sectional Study in General Northeastern Chinese Residents

Yuyan Liu [1], Yongfang Li [1], Jing He [2], Ping Ma [2], Luyang Yu [2], Quanmei Zheng [1] and Guifan Sun [1,*]

[1] Research Center of Environmental and Non-communicable Disease, School of Public Health, China Medical University, Shenyang 110122, China; liuyycmu@163.com (Y.L.); liyongfang_17@163.com (Y.L.); zhengqm2006@163.com (Q.Z.)

[2] Department of Non-Communicable Disease, Shenhe Center for Disease Control and Prevention, Shenyang 110122, China; jinghe18@126.com (J.H.); 18604100018@163.com (P.M.); ylyshcdc@163.com (L.Y.)

* Correspondence: guifan_sun@163.com; Tel.: +86-024-23261744

Table S1. Baseline Characteristics of Participants in Different Age Subgroups (2,336 participants, aged 20 to 94 years old, examined in 2015).

	Men (n=767)				Women (n=1,569)			
	<40 years old (n=40)	40-65 years old (n=473)	≥65 years old (n=254)	*p*-value for trend	<40 years old (n=116)	40-65 years old (n=1,023)	≥65 years old (n=430)	*p*-value for trend
Age (years old)	35.0±3.7	54.7±7.0	70.7±5.2	<0.001	33.8±4.7	55.0±6.8	70.7±5.2	<0.001
BMI (kg/m^2)	25.1±3.0	24.9±3.4	24.4±3.4	0.056	23.0±4.2	24.2±3.2	24.5±3.1	<0.001
SST (mm)	21.2±11.7	24.2±9.5	24.3±8.4	0.214	19.1±10.6	23.8±8.4	24.7±8.4	<0.001
TST (mm)	21.9±13.0	25.7±11.4	26.2±10.4	0.088	20.6±10.5	25.2±9.9	25.0±9.6	0.012
SBP (mmHg)	123.7±8.5	131.5±12.1	133.7±12.1	<0.001	113.9±11.3	126.3±13.9	134.1±13.4	<0.001
DBP (mmHg)	78.5±7.1	81.9±8.6	81.2±8.1	0.708	72.0±8.1	77.9±8.4	80.0±7.8	<0.001
Fasting glucose (mmol/L)	5.1±0.6	5.9±1.8	5.9±1.8	0.131	5.1±0.6	5.7±2.8	6.0±3.0	0.004
TG (mmol/L)	2.0±1.3	2.5±8.0	1.6±1.1	0.175	1.5±1.4	1.8±1.2	1.8±1.0	0.020
TC (mmol/L)	4.8±1.3	5.1±1.0	4.9±0.9	0.130	4.5±0.9	5.3±1.2	5.4±1.1	<0.001
HDL-C (mmol/L)	1.4±0.6	1.4±0.4	1.4±0.4	0.903	1.5±0.5	1.5±0.6	1.5±0.4	0.417
Hypertension (%)	7.5	42.5	48.4	<0.001	6.9	31.1	54.0	<0.001
Smoking (%)				<0.001				0.023
Current	22.5	30.2	13.8		2.6	1.2	2.1	
Past	17.5	15.6	16.6		0.9	1.6	3.3	
Never	60.0	54.1	69.6		96.5	97.3	94.6	
Drinking (%)				<0.001				<0.001
Current	62.5	59.0	35.4		27.6	16.8	4.2	
Past	-	2.5	3.5		-	1.0	0.7	
Never	37.5	38.5	61.0		72.4	82.2	95.1	

Values are presented as mean±SD, or %. **Abbreviations:** BMI: body mass index; SST: subscapular skinfold thickness; TST: tricep skinfold thickness; SBP: systolic blood pressure; DBP: diastolic blood pressure; TG: triglycerides; TC: total cholesterol; HDL-C: high density lipid cholesterol.

Table S2. Associations of Systolic Blood Pressure with All Risk Factors (1,760 participants without anti-hypertensive medication, aged 20 to 94 years old, examined in 2015)

	Men (n=554)			Women (n=1,206)		
	SBP (mmHg)	95%CI	*p*-value	SBP (mmHg)	95%CI	*p*-value
SST Model						
SST (mm)	0.13	0.04, 0.22	0.006	0.23	0.14, 0.31	<0.001
Age (years old)	0.15	0.07, 0.23	<0.001	0.40	0.34, 0.46	<0.001
Fasting glucose (mmol/L)	0.35	-0.12, 0.84	0.149	0.08	-0.15, 0.32	0.491
TG (mmol/L)	0.01	-0.10, 0.13	0.828	0.20	-0.44, 0.85	0.538
TC (mmol/L)	0.63	-0.28, 1.54	0.177	0.41	-0.22, 1.04	0.205
HDL-C (mmol/L)	0.50	-1.66, 2.67	0.649	0.12	-1.26, 1.50	0.867
Smoking (%)						
Never	-	-	-	-	-	-
Past	1.65	-0.79, 4.08	0.185	-1.83	-7.10, 3.45	0.496
Current	0.99	-1.14, 3.12	0.360	1.31	-4.52, 7.14	0.660
Drinking (%)						
Never	-	-	-	-	-	-
Past	1.62	-4.23, 7.48	0.586	2.58	-6.71, 11.87	0.586
Current	1.29	-0.56, 3.15	0.170	-1.28	-3.30, 0.73	0.212
TST Model						
TST (mm)	0.08	0.00, 0.16	0.040	0.13	0.06, 0.21	<0.001
Age (years old)	0.15	0.07, 0.22	<0.001	0.40	0.34, 0.47	<0.001
Fasting glucose (mmol/L)	0.37	-0.11, 0.86	0.129	0.07	-0.16, 0.31	0.546
TG (mmol/L)	0.02	-0.10, 0.13	0.770	0.20	-0.45, 0.85	0.554
TC (mmol/L)	0.72	-0.19, 1.63	0.121	0.48	-0.15, 1.12	0.137
HDL-C (mmol/L)	0.35	-1.81, 2.52	0.748	0.11	-1.28, 1.50	0.874
Smoking (%)						
Never	-	-	-	-	-	-
Past	1.70	-0.75, 4.14	0.174	-1.90	-7.22, 3.41	0.483
Current	1.10	-1.03, 3.23	0.311	1.02	-4.85, 6.90	0.732
Drinking (%)						
Never	-	-	-	-	-	-
Past	1.33	-4.54, 7.20	0.657	2.69	-6.66, 12.04	0.572
Current	1.16	-0.70, 3.02	0.220	-1.25	-3.28, 0.78	0.228

SST and TST were analyzed in separate regression models. **Abbreviations:** SBP: systolic blood pressure; SST: subscapular skinfold thickness; TST: tricep skinfold thickness; 95%CI: 95% confidence interval; BMI: body mass index; TG: triglycerides; TC: total cholesterol; HDL-C: high density lipid cholesterol.

Table S3. Associations of Diastolic Blood Pressure with All Risk Factors (1,760 participants without anti-hypertensive medication, aged 20 to 94 years old, examined in 2015)

	Men (n=554)			Women (n=1,206)		
	DBP (mmHg)	95%CI	*p*-value	DBP (mmHg)	95%CI	*p*-value
SST Model						
SST (mm)	0.03	-0.04, 0.10	0.390	0.10	0.05, 0.15	<0.001
Age (years old)	0.01	-0.04, 0.07	0.682	0.15	0.12, 0.19	<0.001
Fasting glucose (mmol/L)	0.24	-0.12, 0.60	0.191	-0.16	-0.31, -0.02	0.023
TG (mmol/L)	-0.05	-0.13, 0.04	0.286	0.24	-0.16, 0.63	0.235
TC (mmol/L)	0.30	-0.39, 0.98	0.393	0.36	-0.02, 0.75	0.066
HDL-C (mmol/L)	-0.51	-2.12, 1.11	0.539	-0.36	-1.20, 0.48	0.407
Smoking (%)						
Never	-	-	-	-	-	-
Past	0.80	-1.02, 2.62	0.387	-1.02	-4.23, 2.20	0.536
Current	1.26	-0.34, 2.85	0.122	2.45	-1.10, 6.01	0.175
Drinking (%)						
Never	-	-	-	-	-	-
Past	1.90	-2.48, 6.28	0.395	-0.98	-6.64, 4.68	0.734
Current	1.16	-0.22, 2.55	0.099	-0.03	-1.26, 1.20	0.960
TST Model						
TST (mm)	0.01	-0.05, 0.06	0.785	0.04	-0.00, 0.09	0.064
Age (years old)	0.01	-0.05, 0.07	0.673	0.16	0.12, 0.20	<0.001
Fasting glucose (mmol/L)	0.25	-0.11, 0.61	0.174	-0.17	-0.31, -0.03	0.021
TG (mmol/L)	-0.05	-0.13, 0.04	0.292	0.25	-0.15, 0.64	0.221
TC (mmol/L)	0.33	-0.35, 1.01	0.341	0.41	0.02, 0.80	0.039
HDL-C (mmol/L)	-0.56	-2.17, 1.06	0.498	-0.35	-1.19, 0.50	0.421
Smoking (%)						
Never	-	-	-	-	-	-
Past	0.79	-1.04, 2.61	0.398	-0.98	-4.21, 2.26	0.553
Current	1.30	-0.29, 2.89	0.110	2.42	-1.15, 6.00	0.184
Drinking (%)						
Never	-	-	-	-	-	-
Past	1.83	-2.55, 6.21	0.412	-0.92	-6.61, 4.77	0.751
Current	1.13	-0.25, 2.52	0.108	0.03	-1.21, 1.26	0.967

SST and TST were analyzed in separate regression models. **Abbreviations:** DBP: diastolic blood pressure; SST: subscapular skinfold thickness; TST: tricep skinfold thickness; 95%CI: 95% confidence interval; BMI: body mass index; TG: triglycerides; TC: total cholesterol; HDL-C: high density lipid cholesterol.

Table S4. Associations of Hypertension with All Risk Factors (2,336 participants without anti-hypertensive medication, aged 20 to 94 years old, examined in 2015)

	Men (n=767)			Women (n=1,569)		
	OR	95%CI	*p*-value	OR	95%CI	*p*-value
SST Model						
SST (mm)	1.02	1.00, 1.03	0.064	1.05	1.04, 1.07	<0.001
Age (years old)	1.04	1.03, 1.06	<0.001	1.06	1.05, 1.08	<0.001
Fasting glucose (mmol/L)	1.09	1.00, 1.18	0.060	1.01	0.97, 1.05	0.660
TG (mmol/L)	0.99	0.95, 1.03	0.552	1.09	0.99, 1.20	0.083
TC (mmol/L)	1.23	1.05, 1.45	0.011	1.05	0.95, 1.17	0.357
HDL-C (mmol/L)	0.86	0.59, 1.26	0.447	0.79	0.60, 1.04	0.093
Smoking (%)						
Never	-	-	-	-	-	-
Past	0.77	0.50, 1.20	0.240	0.77	0.35, 1.73	0.532
Current	0.81	0.55, 1.19	0.282	0.92	0.36, 2.35	0.863
Drinking (%)						
Never	-	-	-	-	-	-
Past	1.89	0.75, 4.74	0.176	3.24	1.01, 10.37	0.048
Current	1.61	1.16, 2.25	0.005	0.83	0.58, 1.19	0.314
TST Model						
TST (mm)	1.01	0.99, 1.02	0.492	1.04	1.03, 1.05	<0.001
Age (years old)	1.04	1.03, 1.06	<0.001	1.07	1.05, 1.08	<0.001
Fasting glucose (mmol/L)	1.09	1.00, 1.19	0.043	1.01	0.97, 1.05	0.662
TG (mmol/L)	0.99	0.95, 1.03	0.557	1.09	0.99, 1.20	0.089
TC (mmol/L)	1.25	1.06, 1.47	0.008	1.06	0.96, 1.17	0.282
HDL-C (mmol/L)	0.85	0.58, 1.24	0.386	0.79	0.60, 1.04	0.092
Smoking (%)						
Never	-	-	-	-	-	-
Past	0.76	0.49, 1.18	0.227	0.72	0.33, 1.59	0.419
Current	0.82	0.56, 1.20	0.302	0.87	0.35, 2.16	0.758
Drinking (%)						
Never	-	-	-	-	-	-
Past	1.79	0.72, 4.49	0.213	2.95	0.92, 9.44	0.069
Current	1.60	1.15, 2.23	0.005	0.84	0.59, 1.21	0.350

SST and TST were analyzed in separate regression models. **Abbreviations:** SST: subscapular skinfold thickness; TST: tricep skinfold thickness; OR: odds ratio; 95%CI: 95% confidence interval; BMI: body mass index; TG: triglycerides; TC: total cholesterol; HDL-C: high density lipid cholesterol.

Table S5. Crude and Adjusted Associations of Blood Pressure with Per SD Increase of Skinfold Thickness In Men of Different Age Subgroups (554 participants without anti-hypertensive medication, aged 20 to 94 years old, examined in 2015)

	<40 years old (n=39)		40-65 years old (n=349)		⩾65 years old (n=166)		*p*-value for interaction
	BP (mmHg)	95%CI	BP (mmHg)	95%CI	BP (mmHg)	95%CI	
SBP							
Unadjusted Model							
SST	0.03	-2.04, 2.10	1.54*	0.52, 2.56	0.94	-0.73, 2.61	0.868
TST	0.31	-1.87, 2.49	1.21*	0.24, 2.19	0.26	-1.32, 1.84	0.538
Age-adjusted Model							
SST	0.03	-2.07, 2.14	1.51*	0.50, 2.54	0.92	-0.75, 2.59	0.837
TST	0.30	-1.91, 2.52	1.17*	0.19, 2.14	0.23	-1.36, 1.82	0.581
Multi-adjusted Model							
SST	-0.23	-2.78, 2.31	1.53*	0.48, 2.58	0.54	-1.18, 2.27	0.716
TST	0.26	-2.52, 3.04	1.16*	0.16, 2.16	-0.13	-1.78, 1.51	0.707
DBP							
Unadjusted Model							
SST	-0.36	-2.15, 1.44	0.29	-0.50, 1.09	0.74	-0.35, 1.83	0.370
TST	-0.21	-2.11, 1.68	0.16	-0.60, 0.92	0.22	-0.82, 1.25	0.851
Age-adjusted Model							
SST	-0.35	-2.17, 1.47	0.31	-0.49, 1.10	0.70	-0.39, 1.78	0.393
TST	-0.22	-2.14, 1.70	0.19	-0.57, 0.95	0.16	-0.88, 1.19	0.913
Multi-adjusted Model							
SST	-0.56	-2.71, 1.59	0.28	-0.54, 1.01	0.73	-0.39, 1.84	0.313
TST	-0.29	-2.64, 2.08	0.11	-0.66, 0.89	0.17	-0.90, 1.23	0.712

SST and TST were analyzed in separate regression models. Multi-adjusted model: adjusted for smoking, alcohol drinking, fasting glucose, TG, TC, and HDL-C in addition to age-adjusted model. Abbreviations: BP: blood pressure; 95%CI: 95% confidence interval; SBP: systolic blood pressure; DBP: diastolic blood pressure; SST: subscapular skinfold thickness; TST: tricep skinfold thickness. *: *p*-value <0.05; **: *p*-value <0.001

Table S6. Crude and Adjusted Associations of Blood Pressure with Per SD Increase of Skinfold Thickness In Women of Different Age Subgroups (1,206 participants without anti-hypertensive medication, aged 20 to 94 years old, examined in 2015)

	<40 years old (n=114)		40-65 years old (n=816)		≥65 years old (n=276)		*p*-value of interaction
	BP (mmHg)	95%CI	BP (mmHg)	95%CI	BP (mmHg)	95%CI	
SBP							
Unadjusted Model							
SST	1.39	-0.33, 3.11	2.14**	1.18, 3.09	2.96**	1.35, 4.57	0.323
TST	1.03	-1.02, 3.08	1.58*	0.62, 2.55	2.27*	0.71, 3.83	0.470
Age-adjusted Model							
SST	1.40	-0.30, 3.10	1.99**	1.06, 2.92	2.96**	1.35, 4.57	0.223
TST	0.97	-1.06, 3.00	1.25*	0.30, 2.20	2.30*	0.74, 3.86	0.278
Multi-adjusted Model							
SST	0.68	-1.13, 2.49	1.91**	0.96, 2.86	3.26**	1.59, 4.93	0.148
TST	-0.33	-2.59, 1.92	1.26*	0.29, 2.22	2.43*	0.81, 4.06	0.192
DBP							
Unadjusted Model							
SST	1.03	-0.17, 2.24	1.17**	0.60, 1.75	0.83	-0.14, 1.80	0.536
TST	0.92	-0.52, 2.35	0.68*	0.10, 1.26	0.45	-0.48, 1.39	0.410
Age-adjusted Model							
SST	1.04	-0.14, 2.22	1.11**	0.55, 1.68	0.83	-0.13, 1.79	0.618
TST	0.86	-0.54, 2.26	0.54	-0.04, 1.11	0.42	-0.50, 1.35	0.533
Multi-adjusted Model							
SST	0.29	-0.84, 1.43	1.01**	0.43, 1.58	0.86	-0.13, 1.84	0.925
TST	-0.34	-1.76, 1.08	0.47	-0.12, 1.06	0.48	-0.47, 1.43	0.885

SST and TST were analyzed in separate regression models. Multi-adjusted model: adjusted for smoking, alcohol drinking, fasting glucose, TG, TC, and HDL-C in addition to age-adjusted model. Abbreviations: BP: blood pressure; 95%CI: 95% confidence interval; SBP: systolic blood pressure; DBP: diastolic blood pressure; SST: subscapular skinfold thickness; TST: tricep skinfold thickness. *: *p*-value <0.05; **: *p*-value <0.001

Table S7. Crude and Adjusted Associations of Hypertension with Per SD Increase of Skinfold Thickness In Men of Different Age Subgroups (767 participants, aged 20 to 94 years old, examined in 2015)

	<40 years old (n=40)		40-65 years old (n=473)		⩾65 years old (n=254)		*p*-value of interaction
	OR	95%CI	OR	95%CI	OR	95%CI	
Unadjusted Model							
SST	1.16	0.47, 2.87	1.10	0.92, 1.30	1.34*	1.02, 1.75	0.447
TST	1.45	0.58, 3.60	1.08	0.92, 1.28	1.02	0.80, 1.31	0.349
Age-adjusted Model							
SST	1.16	0.47, 2.87	1.10	0.93, 1.31	1.37*	1.05, 1.80	0.371
TST	1.45	0.58, 3.61	1.07	0.90, 1.27	1.05	0.82, 1.35	0.496
Multi-adjusted Model							
SST	-	-	1.08	0.91, 1.30	1.29	0.97, 1.72	0.356
TST	-	-	1.05	0.88, 1.25	1.01	0.77, 1.31	0.548

SST and TST were analyzed in separate regression models. Multi-adjusted model: adjusted for smoking, alcohol drinking, fasting glucose, TG, TC, and HDL-C in addition to age-adjusted model. Abbreviations: OR: odds ratio; 95%CI: 95% confidence interval; SST: subscapular skinfold thickness; TST: tricep skinfold thickness. *: *p*-value <0.05; **: *p*-value <0.001

Table S8. Crude and Adjusted Associations of Hypertension with Per SD Increase of Skinfold Thickness In Men of Different Age Subgroups (1,569 participants, aged 20 to 94 years old, examined in 2015)

	<40 years old (n=116)		40-65 years old (n=1,023)		⩾65 years old (n=430)		*p*-value of interaction
	OR	95%CI	OR	95%CI	OR	95%CI	
Unadjusted Model							
SST	1.36	0.77, 2.41	1.60**	1.38, 1.86	1.59**	1.28, 1.99	0.848
TST	1.13	0.56, 2.30	1.62**	1.40, 1.87	1.39*	1.12, 1.73	0.482
Age-adjusted Model							
SST	1.41	0.78, 2.54	1.61**	1.38, 1.89	1.59**	1.28, 1.99	0.790
TST	1.15	0.56, 2.37	1.58**	1.36, 1.84	1.39*	1.13, 1.73	0.717
Multi-adjusted Model							
SST	1.14	0.60, 2.17	1.60**	1.36, 1.87	1.59**	1.26, 1.99	0.736
TST	0.79	0.34, 1.85	1.58**	1.35, 1.84	1.36*	1.09, 1.69	0.673

SST and TST were analyzed in separate regression models. Multi-adjusted model: adjusted for smoking, alcohol drinking, fasting glucose, TG, TC, and HDL-C in addition to age-adjusted model. Abbreviations: OR: odds ratio; 95%CI: 95% confidence interval; SST: subscapular skinfold thickness; TST: tricep skinfold thickness. *: *p*-value <0.05; **: *p*-value <0.001

Table S9. Blood Pressure and Hypertension in Different Skinfold Thickness Stratified Groups (2,336 Participants, aged 20 to 94 years old, examined in 2015)

	Total number	SBP (mmHg)	DBP (mmHg)	Hypertension (%)	OR (95%CI)
Men	767				
SST<24.0mm	377	127.7±9.8	79.4±7.6	40.1	ref.
SST⩾24.0mm	390	130.3±10.5*	80.2±7.4	45.1	1.15 (0.85, 1.55)
TST<25.0mm	379	128.4±10.2	79.8±7.5	41.4	ref.
TST⩾25.0mm	388	129.6±10.3	79.8±7.5	43.8	1.03 (0.77, 1.39)
Women	1,569				
SST<23.0mm	747	122.0±12.7	75.3±7.6	26.9	ref.
SST⩾23.0mm	822	126.9±14.0**	77.9±8.2**	43.2**	1.88 (1.50, 2.35)**
TST<24.0mm	766	123.2±12.6	76.0±7.7	28.9	ref.
TST⩾24.0mm	803	125.7±14.5*	77.2±8.3*	42.0**	1.65 (1.32, 2.06)**

The mean and standard deviation of blood pressure were estimated using participants without anti-hypertensive medication; SBP and DBP were presented as mean±SD; odds ratio of hypertension was obtained using multivariable logistic linear regression adjusted for smoking, alcohol drinking, fasting glucose, TG, TC, and HDL-C. **Abbreviations:** SBP: systolic blood pressure; DBP: diastolic blood pressure; OR: odds ratio; 95%CI: 95% confidence interval; SST: subscapular skinfold thickness; TST: tricep skinfold thickness. *: *p*-value <0.05; **: *p*-value <0.001
